# Indian Signatures in the Westernmost Edge of the European Romani Diaspora: New Insight from Mitogenomes

**DOI:** 10.1371/journal.pone.0075397

**Published:** 2013-10-15

**Authors:** Alberto Gómez-Carballa, Jacobo Pardo-Seco, Laura Fachal, Ana Vega, Miriam Cebey, Nazareth Martinón-Torres, Federico Martinón-Torres, Antonio Salas

**Affiliations:** 1 Unidade de Xenética, Departamento de Anatomía Patolóxica e Ciencias Forenses, and Instituto de Ciencias Forenses, Grupo de Medicina Xenómica (GMX), Facultade de Medicina, Universidade de Santiago de Compostela, Galicia, Spain; 2 Grupo de Investigación en Genética, Vacunas, Infecciones y Pediatría (GENVIP), Hospital Clínico Universitario and Universidade de Santiago de Compostela (USC), Galicia, Spain; 3 Fundación Pública Galega de Medicina Xenómica-SERGAS, Grupo de Medicina Xenómica-USC, IDIS, Santiago de Compostela, Galicia, Spain; 4 Pediatric Emergency and Critical Care Division, Department of Pediatrics, Hospital Clínico Universitario de Santiago, Santiago de Compostela, Galicia, Spain; University of Perugia, Italy

## Abstract

In agreement with historical documentation, several genetic studies have revealed ancestral links between the European Romani and India. The entire mitochondrial DNA (mtDNA) of 27 Spanish Romani was sequenced in order to shed further light on the origins of this population. The data were analyzed together with a large published dataset (mainly hypervariable region I [HVS-I] haplotypes) of Romani (*N* = 1,353) and non-Romani worldwide populations (*N*>150,000). Analysis of mitogenomes allowed the characterization of various Romani-specific clades. M5a1b1a1 is the most distinctive European Romani haplogroup; it is present in all Romani groups at variable frequencies (with only sporadic findings in non-Romani) and represents 18% of their mtDNA pool. Its phylogeographic features indicate that M5a1b1a1 originated 1.5 thousand years ago (kya; 95% CI: 1.3–1.8) in a proto-Romani population living in Northwest India. U3 represents the most characteristic Romani haplogroup of European/Near Eastern origin (12.4%); it appears at dissimilar frequencies across the continent (Iberia: ∼31%; Eastern/Central Europe: ∼13%). All U3 mitogenomes of our Iberian Romani sample fall within a new sub-clade, U3b1c, which can be dated to 0.5 kya (95% CI: 0.3–0.7); therefore, signaling a lower bound for the founder event that followed admixture in Europe/Near East. Other minor European/Near Eastern haplogroups (e.g. H24, H88a) were also assimilated into the Romani by introgression with neighboring populations during their diaspora into Europe; yet some show a differentiation from the phylogenetically closest non-Romani counterpart. The phylogeny of Romani mitogenomes shows clear signatures of low effective population sizes and founder effects. Overall, these results are in good agreement with historical documentation, suggesting that cultural identity and relative isolation have allowed the Romani to preserve a distinctive mtDNA heritage, with some features linking them unequivocally to their ancestral Indian homeland.

## Introduction

There are about 8–10 million Romani in Europe; the majority of them live in Central and Southeastern Europe [1]. An Asian origin for the Roma has been proposed several times on the basis of linguistic, cultural, and genetic evidence. Most historical information on the Romani has been provided by neighboring populations, but the written record is very limited. According to Ioviţă and Schurr [2], the ‘Gypsy-lorist’ paradigm provides the most popular explanation for their origin: the Romani are itinerant peoples originating from a single population group coming from South Asia, preserving their cultural identity over generations with little exchange with surrounding populations [2]. Although this paradigm is problematic when considering the ethno-historical and linguistic context of the Romani in Europe, there is growing evidence showing a common biological origin for the Romani [2]. Most historians agree that the Romani originated in India, and that they migrated to Europe at some point between the 5–10^th^ century AD [3]. Comparative linguistics has suggested that Northwest Indian languages, such as Punjabi or Kashmiri are the most related to Romani [4]. Initially, their ancestors reached the Balkans and settled preferentially at different locations within Eastern and Central Europe, including present-day Romania, Bulgaria, Macedonia, Hungary, and the Slovak Republic; but they were soon widely dispersed in Europe [2]. Romani migration to North America began in colonial times; large-scale trans-Atlantic migrations occurred during the 19^th^ century, preferentially to the USA (from Great Britain) and Brazil.

The Romani have been described as a conglomerate of genetically isolated founder populations [5]. Their demographic history provides a good explanation for the high incidence of several rare genetic Mendelian disorders and private mutations compared to other neighboring European populations [6]. Molecular anthropological studies have provided new relevant insight into the demographic history of this population. Thus, analyses of the Y-chromosome and mtDNA have revealed the existence of interesting genetic features in the Romani [7]–[9]. Kalaydjieva et al. [10] analyzed three groups of Vlax Roma from Bulgaria and identified a close mtDNA and Y-chromosome resemblance between these groups, most likely indicating a common and recent origin. Gresham et al. [8] analyzed the Romani population mainly residing in Bulgaria and reported a high frequency of the Y-chromosome lineages defined by a mutation in the locus M82 (identifying the Indian specific Y-chromosome haplogroup H1a1a-M82). They also observed a high frequency of the Asian mtDNA macro-haplogroup M with little genetic variation within these populations; according to these authors, this pattern would be consistent with a small group of founders splitting from a single ethnic population most likely located in the Indian subcontinent. In a follow-up study [11], new aspects of the Vlax Roma were revealed, such as the existence of recent splits occurring after their arrival in Europe, asymmetric migration flows for males and unequal growth rates. Malyarchuk et al. [12] analyzed the control region segment of a sample of Polish Roma, and the mitogenome of a Polish donor carrying a M5 Roma lineage; their study also indicated the existence of mtDNA founder effects in the Polish Roma. Over the last few years, other Romani populations have been analyzed, including individuals from Hungary, Romania, Slovakia and Poland [13] [14] [15] [16] [17]. Regarding uniparental markers, a recent Y-chromosome study by Regueiro et al. [18] claims that the Roma descended from southern Indian populations, thereby contradicting various reports based on mtDNA and autosomal studies (see below) that pointed to northwest India as the homeland of proto-Romani.

The most recent genome-wide SNP study on European Romani by Mendizabal et al. [19] indicated that their diaspora occurred from a single initial founder population from North/northwest India ∼1.5 thousand years ago (kya), followed by a rapid migration through the Near or Middle East, and then, about 0.9 kya, through the Balkans to Western Europe. Almost at the same time, Moorjani et al. [20] analyzed genome-wide SNPs from 27 Roma samples belonging to six European groups; their data indicate an 80% Western European ancestry and that the admixture of South Asian and European ancestry occurred about 0.85 kya.

Very few studies have been carried out in Iberian Romani. Fernández et al. [21] analyzed the prevalence of mtDNA haplogroups and HLA class II among southern Spanish Romani suffering from multiple sclerosis, but no statistical association was found between patterns of mtDNA haplogroup frequencies and multiple sclerosis. Mendizabal et al. [22] analyzed individuals from different Roma communities in Portugal and northeast Spain; the patterns observed on the mtDNA mirrored different migration routes with several founder effects along a North/East migration route shared with other Central European Roma. The results suggested the Punjab state in Northwest India as the putative ancestral homeland of the European Roma, which is in agreement with other linguistic and anthropological studies.

Against this background, our analysis of Roma mitogenomes serves two main purposes. Firstly, in previous studies, a Romani ancestry was attributed to certain control region motifs but the level of genetic resolution explored did not allow researchers to allocate Romani lineages to the most up to date global mtDNA phylogeny [23]; mitogenomes, in contrast, represent the maximum level of resolution possible for the mtDNA molecule, allowing a better understanding of Romani-specific lineages. Secondly, mitogenomes can reveal demographic features (including dating of lineages) that may not be possible using partial mtDNA sequences (i.e. HVS-I segments).

## Materials and Methods

### Samples

Blood and saliva samples were collected from a total of 27 self-declared Romani within the framework of ESIGEM (http://www.esigem.org) [24]; all these individuals had suffered from meningococcal disease. Written informed consent was obtained from all patients. Genetic analysis of the individuals in the present study was approved by the Comité Ético de Investigación Clínica de Galiza (CEIC; Servicio Galego de Saúde, Galicia, Spain). The study conforms to the Spanish Law for Biomedical Research (Law 14/2007- 3rd of July). All samples were genotyped for 600,000 autosomal SNPs (authors' unpublished data); these data represent a small subset of a large SNP dataset of a Spanish meningococcal disease cohort. Another subset of this cohort was used in a mtDNA case-control study on meningococcal disease where a few mtDNA SNPs were tested in patients against two control sample sets [25]. The autosomal data used in the present study were used exclusively to infer possible close familial relationships.

### PCR amplification and sequencing analysis

PCR amplification was performed in a 9700 Thermocycler (AB) using 32 cycles of amplification and the temperature profile: 95°C for 10 s, 60°C for 30 s, and 72°C for 30 s. Analysis of mitogenomes was carried out as previously described [26], [27]. Mitogenomes have GenBank accession numbers KF055863–KF055889.

### Statistical analysis

The phylogenetic reconstruction of mitogenomes was carried out by building maximum parsimony trees. The time to the most recent common ancestor (TMRCA) for each cluster was calculated by computing the averaged distance (ρ) of all haplotypes in a clade to the respective root haplotype and heuristic estimates of the standard error (σ) were calculated from an estimate of the genealogy [28]. Hotspot mutations such as T16182C, T16183C and T16519C were excluded from the calculations. Mutational distances were converted into years using the corrected evolutionary rate proposed by Soares et al. [29].

Identity by state (IBS) was computed using PLINK [30]. In-house R scripts (http://www.r-project.org) were used to display results obtained from PLINK. GWAS data from non-Romani Spanish individuals were used for comparison.

Mitochondrial DNA data from different Romani populations were collected from the literature (*N* = 1,353 mtDNA profiles in total): 232 from several regions in Bulgaria, 18 from Vilnius in Lithuania, and 25 from Madrid in Spain [8], [10], 384 from the Croatian region of Baranja-Medimurje [17], 205 from the Baranya county in Hungary [13], 69 from Zielona Góra-Nowa Sól in Poland [12], 192 from the Slovak Republic [16], 138 from Portugal and 76 from Barcelona in Spain [22] and 14 from Málaga in Spain [21]. Most of the data consist of of HVS-I sequences, although some studies also reported the HVS-II, coding region SNPs and/or RFLPs. Additional searches for partial mtDNA profiles were carried out in the published literature, the Sorenson database (http://www.smgf.org/), Mitosearch (http://www.mitosearch.org/), and EMPOP (http://empop.org).

Admixture estimates of European Romani with non-European Romani were calculated simply on the basis of phylogeographic data; that is, by estimating the proportion of South Asian lineages among Romani (haplogroup M excluding M1 candidates; M(×M1)). Admixture estimates were previously obtained on mtDNA data based on an algorithm that weighs matched haplotypes existing between the source population (India) and the Romani [22]; however, it is important to note that many South Asian haplotypes existing in the Romani are not present in the source Asian population (although their phylogeographic characteristics point to their undoubtedly Asian origin). For instance, the most characteristic Romani M5a1b1a1 haplotype (the one that matches the motif of M5a1b1a1 and that constitutes 15% of the total mtDNA Romani pool; **Table S2**) of Indian origin is very rare in India (0.001%; [31]). Therefore, as per the algorithm mentioned above, this Indian haplotype would not contribute to the Indian ancestry proportion of the Romani (e.g. similar to other African or European haplotypes).

## Results

### Investigating close familial relationships among Romani

**Figure 1:**
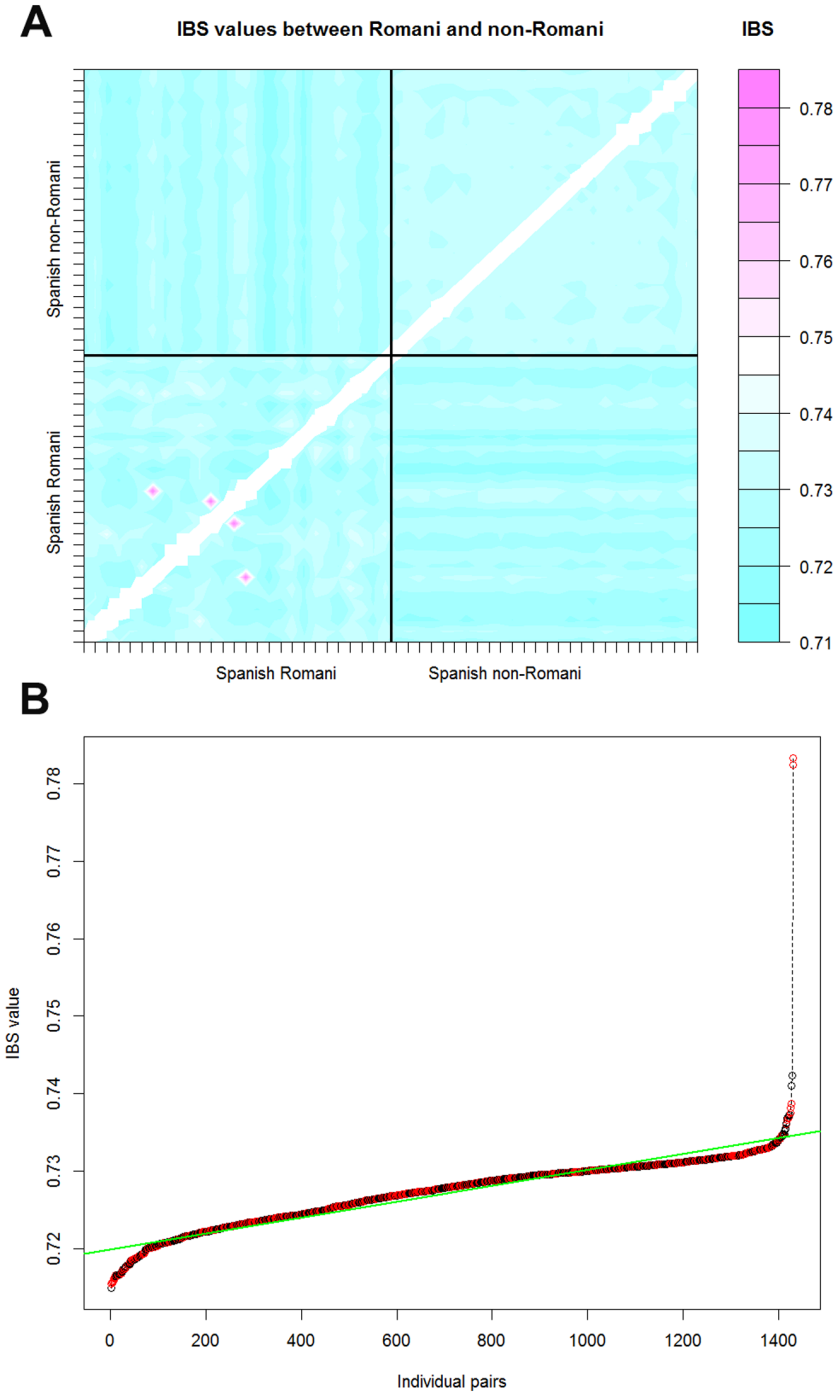
Analysis of IBS carried out on Romani and non-Romani individuals based on genome-wide SNP data. (A) IBS values between Romani and non-Romani individuals. The non-Romani population sample was taken from the same Spanish region where the Romani samples were collected for the present study. The pink dots correspond to the two pairs of Romani individuals showing much higher IBS values than those observed between other Romani or non-Romani individuals. (B) All pairs of IBS values between Romani and non-Romani individuals are sorted from the lowest to the highest; the two highest values on the right of the figure correspond to the two pairs of Romani individuals in Figure 1A.

SNP autosomal data indicate that there are two pairs of Romani individuals that show high IBS values compared to the rest of the Romani and other non-Romani Iberian individuals (**Figure 1**): R831 and V103 (**Table S1**: haplotypes #15 and #16, respectively), and 7E264 and 9B104 (**Table S1**: haplotypes #11 and #27, respectively). IBS values for these two pairs of genetic profiles are compatible with a second degree familial relationship [32] that is statistically significant (in both cases *P*-value<10^−7^) according to the test proposed by Lee [33]. Note, however, that only the former pair of individuals shares a common mtDNA haplotype (**Table S1**). Therefore, it can be considered that the Romani individuals analyzed in the present study (with the exception of one of the two pairs indicated above) are not closely related from the point of view of their maternal lineage. Thus, the fact that several Romani share the same mtDNA haplotypes should be interpreted as owing to a shared and recent demographic history – which is coherent with isolation and low effective population size.

### The ancestral Indian legacy in Iberian Roma

**Figure 2:**
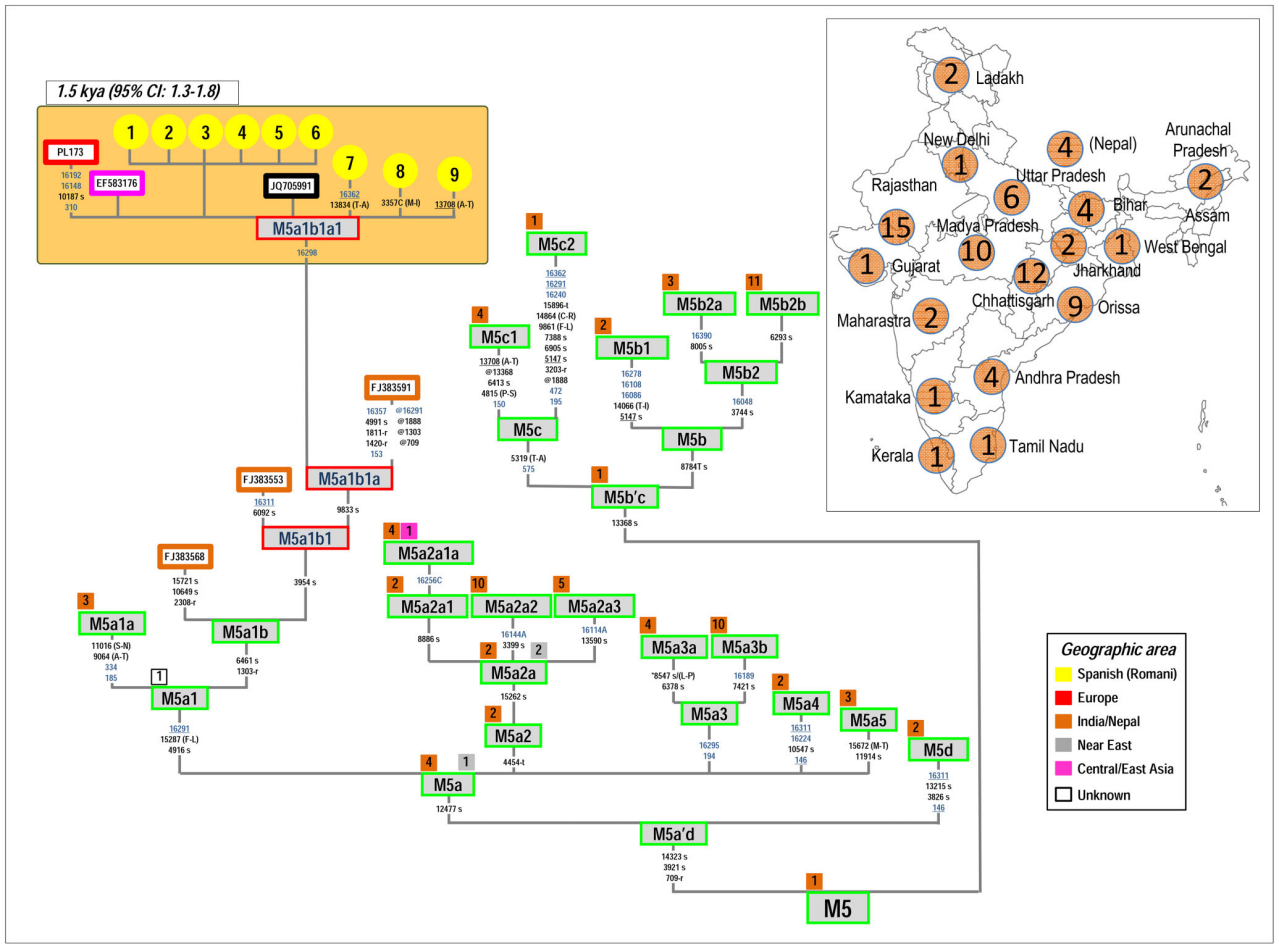
Maximum parsimony tree of haplogroup M5 Romani mitogenomes. The inset map shows the geographic location and sample size of all the M5 genomes observed in India subcontinent. The position of the revised Cambridge reference sequence (rCRS) is indicated for reading sequence motifs [52]. Mitochondrial DNA variants are indicated along the branches of the phylogenetic tree. An asterisk (*) as prefix indicates a position located in an overlapping region shared by two mtDNA genes. Mutations are transitions unless a suffix A, C, G, or T indicates a transversion. Other possible suffixes indicate insertions (+), synonymous substitution (s), mutational changes in tRNA (-t), mutational change in rRNA (-r), non-coding variant located in the mtDNA coding region (-nc) and an amino acid replacement (indicated in round brackets). Variants underlined represent recurrent mutations in this tree while a prefix ‘@’ indicates a back mutation. Mutational hotspot variants at positions 16182, 16183, and 16519, as well as variation around position 310 and length or point heteroplasmies were not considered for the phylogenetic reconstruction. The numbers in small squares attached to the haplogroup labels indicate the number of occurrences (mitogenomes) of the corresponding haplogroups found in public databases; the color of the squares indicates their geographic origin according to the legend inset. Spanish Romani complete genomes obtained in this study are indicated with yellow circles. More details on the geographic or ethnic origin of all the mitogenomes used in this network are provided in **Table S1**. The Indian M5a1b1a genome (FJ383591) seems to belong to M5a1b1a, but note that it lacks four diagnostic sites, most likely due to sequencing or documentation errors [53]–[55].

The most outstanding feature of our Iberian Romani mitogenomes is the high frequency (nine mitogenomes; 33%) of the Southeast Asian haplogroup M5. Kalaydjieva et al. [10] identified this haplogroup in Bulgarian Romani and almost concomitantly it was identified by Gresham et al. [8] in other European Romani (including another population from Bulgaria); however, only the control region motif (the HVS-I segment) could be revealed at that time. Further studies allowed the Romani lineages to be allocated to the sub-branch M5a1b [15], [34]. By collecting all of the M5 mitogenomes available in the literature and databases, together with the new ones generated in the present study, it is now possible to allocate the Romani branch to the global M5 phylogeny (**Figure 2**). The root of M5a1b (identified by coding region motif G1303A-A6461G) is represented by a single haplotype sampled in India (**Figure 2**). Three other mitogenomes from India constitute its sister clade (defined by the sequence motif G185A-T334C-G9064A-G11016A), M5a1a (**Figure 2**). The nine M5a1b genomes observed in our Spanish Romani individuals coupled with six additional genomes available in the literature allowed us to reconstruct the phylogeny of this clade and reveal the particular features of the specific Romani branch (**Figure 2**). There is only one sub-clade within M5a1b, M5a1b1 (characterized by transition C3954T); M5a1b1a is its only nested haplogroup (transition T9833C). Emerging from M5a1b1a is the Romani specific branch, M5a1b1a1, featured by the control region transition T16298C.

Almost al M5 mitogenomes (excluding M5a1b1a1) were sampled in India (**Figure 2**), with only a few exceptions: four in Nepal, one in China, and three in the Near East. Therefore, the phylogeny clearly points to M5 as originating in India. This includes the root of M5a1b1 and the root of its nested sub-branch M5a1b1a (both appear to originate in India, as suggested by the presence of two Indian genomes). On the contrary, the Romani M5a1b1a1 has been found in Europe only: one Russian (GenBank code: EF583176), one Polish (#PL173, [12]), one individual of unknown origin (JQ705991), and nine Spanish Romani (**Figure 2**). Therefore, from the phylogeographic characteristics of the mitogenomes alone, it is not clear if M5a1b1a1 arose originally in India or in some place on the way to Europe, although it appears that all its immediate ancestors and in general haplogroup M5 are from India. The TMRCA of M5a1b1a1 is 1.5 kya (95%CI: 1.3–1.8).

**Figure 3:**
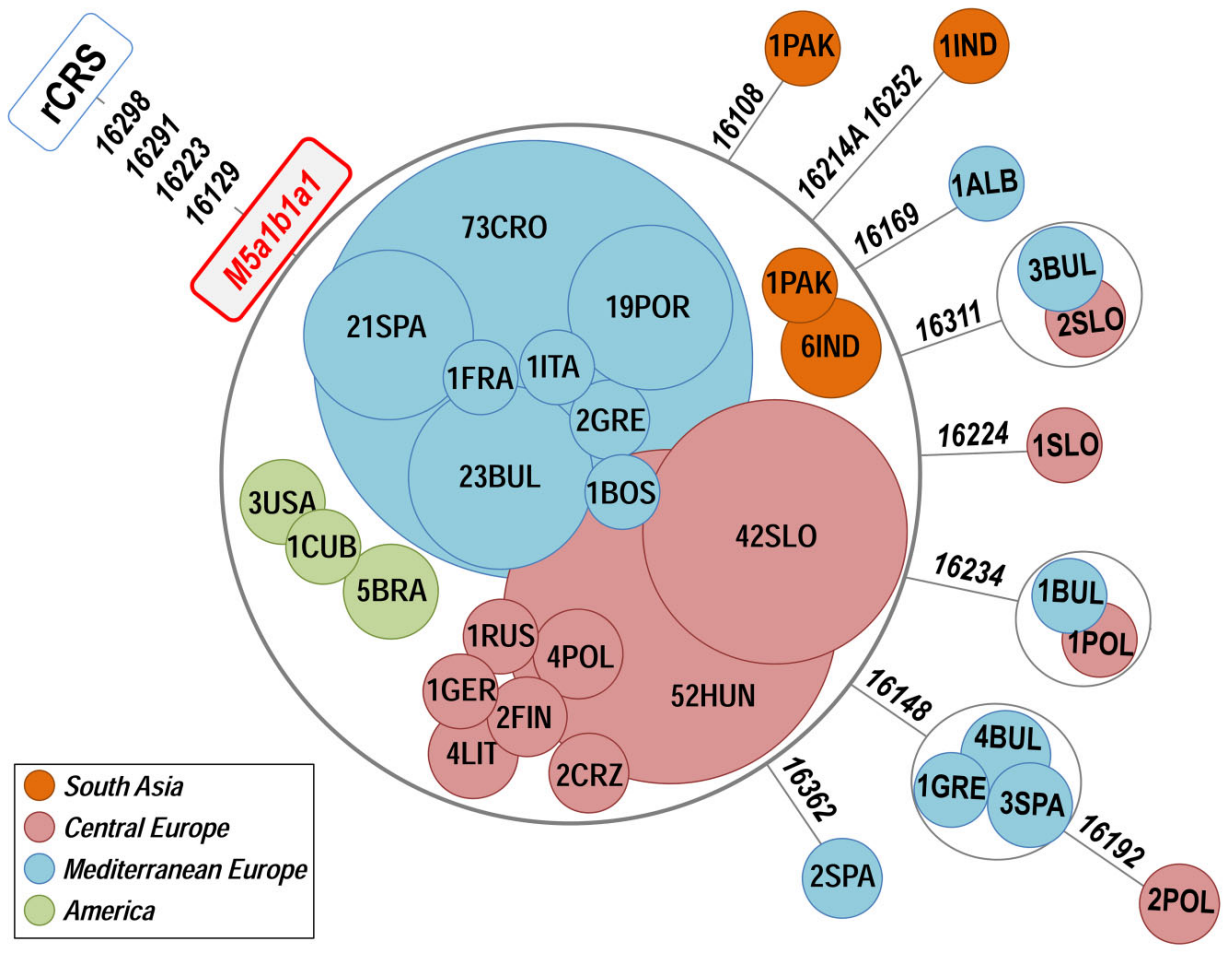
Maximum parsimony tree of M5a1b1a1 HVS-I sequences. Population codes are as follows: ALB = Albania; BOS = Bosnia; BRA = Brazil; BUL = Bulgaria; CRO = Croatia; CRZ = Czech Republic; CUB = Cuba; FIN = Finland; FRA = France; GER = Germany; GRE = Greece; HUN = Hungary; IND = India; ITA = Italy; LIT = Lithuania; PAK = Pakistan; POL = Poland; POR = Portugal; RUS = Russia; SLO = Slovakia; SPA = Spain; USA = United States of America. See **Table S3** for detailed geographic information on these haplotypes. See caption to **Figure 2** for more information on the features of the tree.

**Figure 4:**
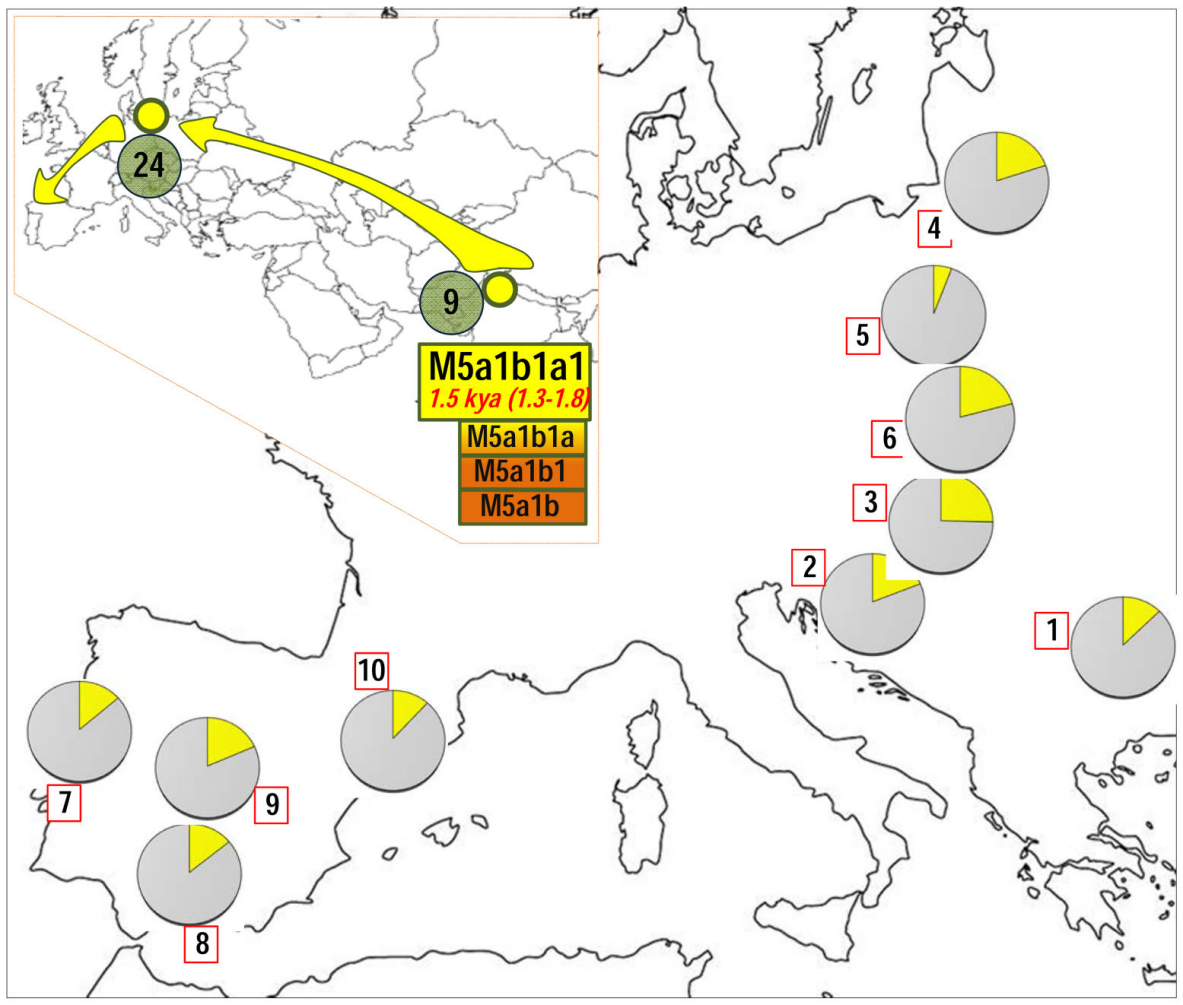
Map showing the frequency of haplogroup M5a1b1a1 control region sequences (pie charts) in different European Romani groups. The inset map represents this clade as ultimately originated in India; the numbers in the green circles represent the occurrences of M5a1b1a in non-Romani individuals in Eurasia (see **Table S3** for references): 24 incidences in Europe and 9 incidences in India. References for the European Romani groups (red squares in the map) are as follows: 1 = Bulgaria [8], [10]; 2 = Croatia [17]; 3 = Hungary [13]; 4 = Lithuania [8]; 5 = Poland [12]; 6 = Slovakia [16]; 7 = Portugal [22]; 8 = Málaga (Southern Spain) [21]; 9 = Madrid (Central Spain) [8]; 10 = Barcelona (Northeastern Spain) [22].

Further phylogeographic information of the Romani clade can be investigated by analyzing control region sequences, which represent a much larger database from all over the world. The control region motif of M5a1b1a1 is defined by positions G16129A-C16223T-C16291T-T16298C; therefore, it can be easily searched in public databases. In a large worldwide database (>150,000 profiles), and including the mtDNAs observed in our sample, we found 291 HVS-I profiles matching this motif (most of them also containing information for HVS-II segments and coding region SNPs and/or RFLPs [and in our samples, the mitogenomes]; **Table S3**). **Figure 3** shows a network of all M5a1b1a1 HVS-I segments (excluding three of unknown geographic origin). The star-like phylogeny of M5a1b1a1 is compatible with its recent age as inferred from the analysis of mitogenomes. A total of 246 M5a1b1a1 HVS-I haplotypes out of 291 (85%) were observed in Romani from Europe (**Table S3**). Thus, this haplogroup is the most frequent among Romani (∼18% on average), but its frequency varies substantially between different Romani groups; e.g. Hungarians (∼25%) [13], Slovakians (∼21%) [16], Croatians (19%) [17], Spanish and Portuguese (∼16%) ([8], [21], [22] and present study), Bulgarians (∼13%) [8], Polish (∼6%) [12] (**Figure 3** and **4**). In Eurasia, and outside Europe, M5a1b1a1 is exclusively found in the Indo-Pakistani region: seven M5a1b1a1 HVS-I haplotypes were observed in India, five of them in the Punjab (Northwest); and two of them in Northeast Pakistan, close to the frontiers with the Punjab. Its geographic distribution, coupled with the global distribution of M5 (**Figure 2**) point to Northwest India as the most likely origin of the M5a1b1a1 haplogroup.

Most of the non-Romani M5a1b1a1 HVS-I mtDNAs were found in those European countries (representing ∼8% of the total European M5a1b1a1 haplotypes; 24/291) where the largest Romani populations live (Slovak Republic, Croatia, Czech Republic, Bulgaria, Spain, etc.); **Figure 4**. This percentage most likely mirrors matrilineal exchange between Romani and non-Romani populations in Europe.

The remaining M5a1b1a1 mtDNAs were found in America (9/291; ∼3%), namely, five Brazilians, three USA citizens, and one Cuban (**Table S3**).

**Figure 5:**
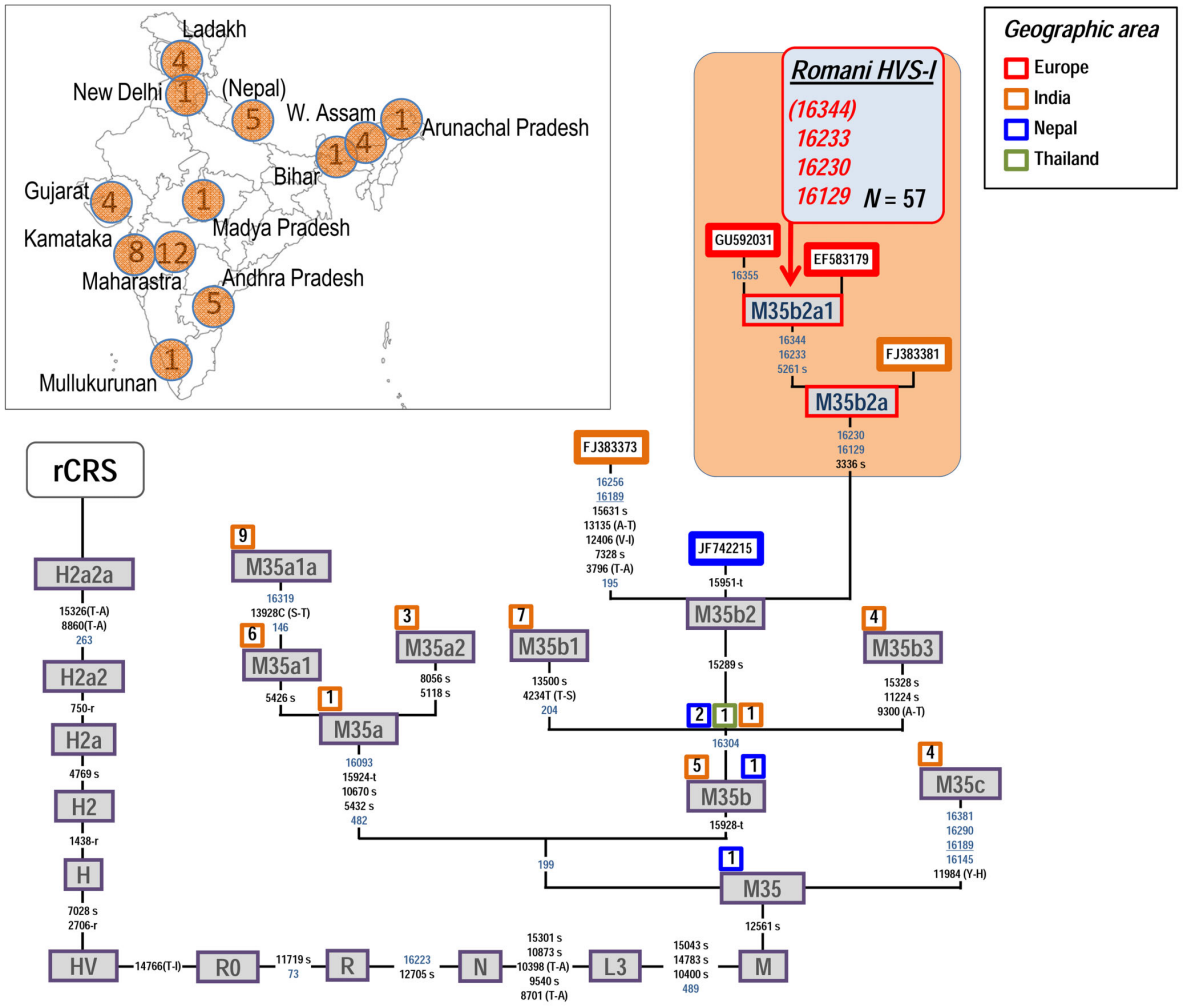
Maximum parsimony tree of haplogroup M35 mitogenomes. The inset map shows the geographic location and sample size of all the M5 genomes observed in the Indian subcontinent. See caption to **Figure 2** for more information on the features of the tree.

The Romani HVS-I database reveals the existence of other South Asian lineages (apart from M5a1b1a1) in the European Romani. M35b (HVS-I motif: C16223T-T16304C) is present in Romani from Central and Eastern Europe (but not in Iberia). Even though this basal motif is not very informative (owing to the high mutation rate of the transition T16304C; [29]) and could be compatible with other haplogroups (Phylotree; http://www.phylotree.org/), almost all Romani share a distinctive M35b HVS-I profile (it alone constitutes ∼4% of all the European Romani; *N* = 57) which is almost absent in non-Romani individuals: G16129A-C16223T-A16230G-C16233T-T16304C-C16344T. By collecting the full set of mitogenomes available in GenBank and public databases (*N* = 50; **Table S1**), we could reconstruct and update the phylogeny of M35 (**Figure 5**). Almost all M35 mitogenomes were sampled in India (*N* = 42) and a few in Nepal (*N* = 5) (**Table S1**). The phylogeny reveals new sub-branches of M35. The above mentioned Romani M35b HVS-I haplotype fits perfectly within one of these new branches, here named as M35b2a1. M35b as a whole is common and seems to originate from North India; the most phylogenetically closely related genome of the Romani M35b2a1 clade (GenBank acc. n°: FJ383381, haplogroup M35b2a; **Table S1**) comes from the Gujarat region in Northwest India. Within the Romani M35b2a1, there are only two mitogenomes, both found in Central Europe (GU592031 and EF583179).

M18 (control region motif: T246C-C16223T-A16318T) is only present in six Spanish Romani; five sampled in Barcelona (Northeast; [22]) and one in Jaén (South; present study) and all carry the additional diagnostic variant T246C at the HVS-II; there is another candidate from Málaga (South; [21]) for which there is not HVS-II data. M18 is completely absent in Central and Eastern European Romani. The great majority of M18 haplotypes are found in Southwest India (where M18 constitutes ∼2% of the lineages; [31]); and few appear also in the Malbars from the Reunion Island, which are known to be mainly of Indian ancestry [31].

### European/Near Eastern lineages in Iberian Romani

**Figure 6:**
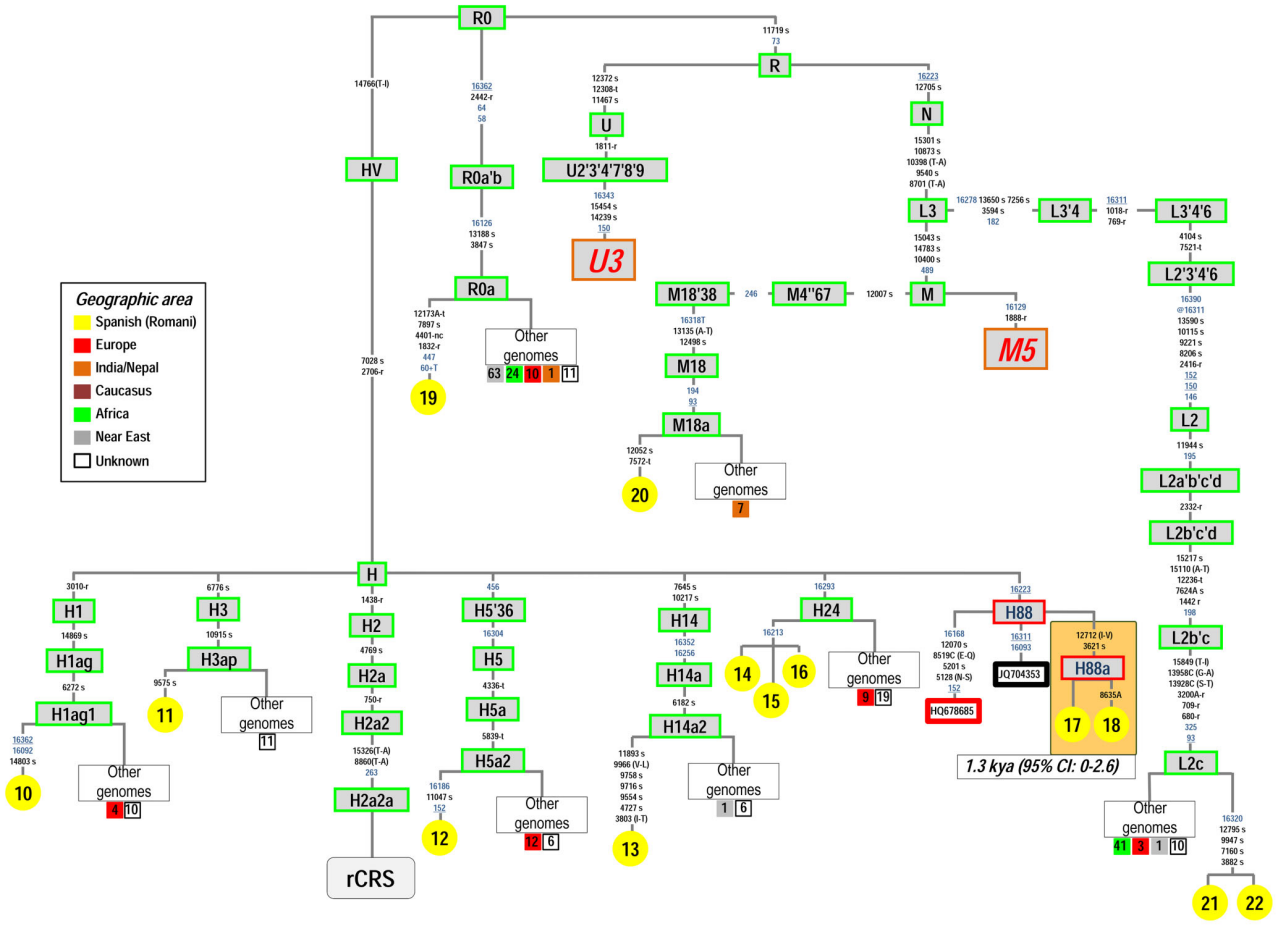
Maximum parsimony tree of the Spanish Romani mitogenomes analyzed in the present study excluding those belonging to haplogroup M5 (**Figure 2**), and U3 (**Figure 7**). See the caption to **Figure 2** for more information on the features of the tree.

A number of new mtDNA branches in the Spanish Romani emerge when contrasted against the worldwide phylogeny. A few of them, however, are represented by single sequences and therefore, we did not assign haplogroup labels to them, while awaiting the discovery of new sequences and confirmation of their haplogroup roots (**Figure 6**).

Within haplogroup H, there are six new sub-branches (**Figure 6**). One of them, H5a2, is characterized by control region transitions T152C-C16186T plus the coding region variant C11047T. By searching worldwide the distinctive control region motif C16186T together with T16304C (diagnostic of haplogroup H5), and disregarding haplogroup T members sharing this motif, we found a strong signal of H5a2 as being almost always present among several Romani populations. However, while this appears to be very rare in Iberia (1/280; ∼0.004%), it has been found at high frequencies in some Bulgarian Romani [8], [10]: Lingurari (*N* = 19; 77%) and the Intreni (*N* = 5; 31%).

There are three identical haplotypes belonging to haplogroup H24 (**Figure 6**). All of them carry the characteristic polymorphism G16213A. The H24 control region motif A16293G (diagnostic of H24) and G16213A (disregarding some compatible sub-Saharan haplogroup L African branches [mostly L1b] and members of the Asian/Native American haplogroup A2) is virtually absent in worldwide databases, including Romani communities. Thus, there are only a few H24 candidates (matching the HVS-I motif): one haplotype is in France (Mitosearch), one haplotype in Álava (North Spain), and one in the USA (EMPOP). This feature, together with the fact that the three carriers of H24 bear identical haplotypes, suggests a very recent origin for this lineage among Iberian Romani.

The two genomes carrying transitions T3621C-A12712G determine a new sub-branch of haplogroup H88 (H88a) (**Figure 6**). A tentative age for this minor haplogroup would be 1.3 kya (95%CI: 0–2.6); therefore, it most likely originated soon after the initial period of the Romani diaspora from India; perhaps in the Middle East or the Caucasus where other sister clades of H88 exist.

Most of the phylogenetic branches of haplogroups H5a2 and H24 and H88 have been found in Europe (**Figure 6**), indicating that these Romani clades were most probably generated recently along their European diaspora.

**Figure 7:**
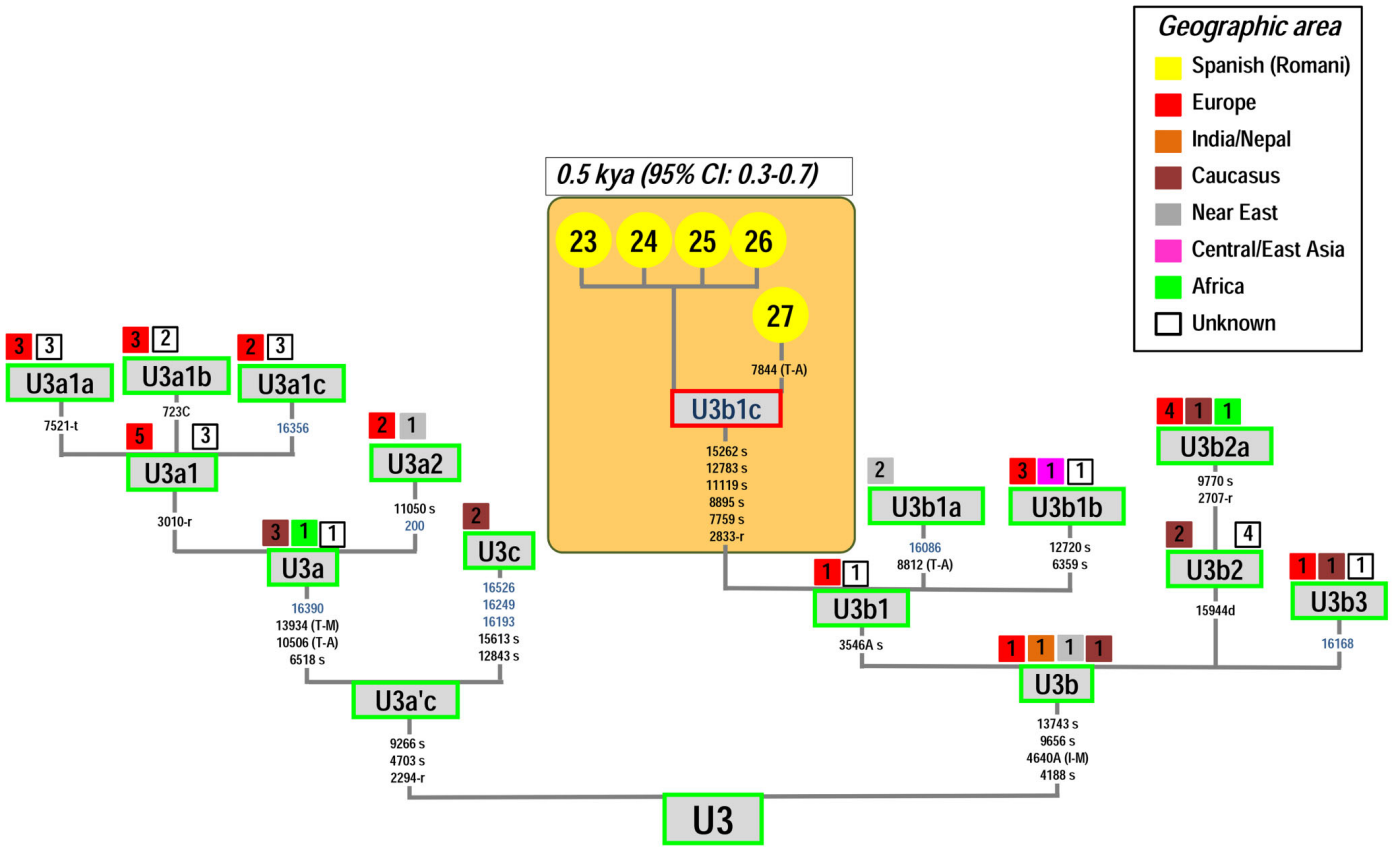
Maximum parsimony tree of haplogroup U3 mitogenomes. See caption to **Figure 2** for more information on the features of the tree.

**Figure 7** shows the most up to date skeleton of the haplogroup U3 phylogeny. U3 is defined by the transition A16343G. It is found in Europe at low frequency (below 1%; [35]) and in the Near East at slightly higher frequency (∼2.5%) [36]. In the Caucasus, U3 reaches frequencies of about 3–4%; but the highest proportion of U3 was found in populations from Iran (e.g. 17.6% in the Lur from Southwestern Iran in the Zagros Mountains) [36]. U3 has also been reported at a high frequency among different Romani groups [8], [22]. There are 62 U3 mitogenomes available. Most of these genomes have been found in individuals sampled in Southeast Asia, the Middle East, and Europe. However, the phylogeographic information for these mitogenomes is very limited. The five U3 Spanish Romani mtDNAs analyzed in the present study all fall within the same sub-branch, named here as U3b1c, which is defined by variants A2833G-T7759C-T8895C-C11119T-T12783C-T15262C. The most closely related lineages of U3b1c were observed in Finland, Iraq, Italy, Portugal, Russia, Slovakia and Yemen. The Romani U3b1c show very limited variability; five of them share exactly the same mtDNA genome, while one of them shows only one transition on top of the basal motif of U3b1c. The TMRCA of U3b1c is 0.5 kya (95% CI: 0.3–0.7). Additional information on Romani U3 can be gathered from HVS-I databases (**Table S2**). The Romani U3 haplotypes available in the literature are all based on control region sequences but were not phylogenetically distinguishable from other U3 branches (although there are some clades that are determined by control region variants within U3; **Figure 7**); therefore, control region data are not very informative from the phylogeographic point of view. U3 constitutes the second most frequent haplogroup in European Romani (12.4%); however, its frequency varies from 7.5% in Central and Eastern European Romani to. 31.4% in Iberian Romani. U3 shows very little variability in this control region database: there are only seven different HVS-I haplotypes in 171 individuals, and the majority (91% of the total U3 haplotypes) bears the basal control region mutation only (A16344G) while the other haplotypes have only up to three step mutations on top of the basal motif (**Table S2**). The low variability observed in this large control region database is in good agreement with the young age of U3 estimated from mitogenomes.

### Sub-Saharan African lineages in Roma

Surprisingly, two of the self-declared Romani carried two typical sub-Saharan African genomes, both belonging to haplogroup L2c (**Figure 6**). They share exactly the same sequence, which might suggest a close relationship between them. Note, however, that high-throughput SNP data allow us to reject such a relationship (see Material and Methods) while confirming their Romani genomic ancestry. L2c is largely confined to West and eastern Central Africa [37] and it has been carried to America mediated by the trans-Atlantic slave trade; thus, L2c can now be found in North, Central, and South America [38]–[41].

We did not found any closely related branch to this Romani L2c lineage; therefore, this could represent a new L2c cluster that has been lost in Africa [42] or is too rare there. In the control region, there is only one diagnostic site, the C16320T transition. When searching an extensive database of control region profiles for the motif A93G-C198T-C325T-C16320T, we only found two candidates: one observed in Angolares (São Tomé and Principe; Equatorial Guinea) [43], and the other in African-Americans [44].

The Romani control region database reveals the existence of five additional sub-Saharan L-haplotypes: four were sampled in the Slovak Republic and one in Hungary (**Table S2**). In addition, there are two identical haplotypes sampled in Portugal belonging to haplogroup M1a1 [45], which could also be of African origin; identical matches to this haplotype can be found in North and East Africa [46]–[48], but also in the Middle East and Saudi Arabia [49].

The sub-Saharan component in European Romani as estimated from the control region Romani database is therefore ∼0.5–0.6%.

## Discussion

Previous attempts to analyze the population genetic background of the European Romani have focused primarily on the mtDNA control region data (mainly HVS-I) and, most recently, on autosomal SNPs at a genome-wide level. The present study focused on the analysis of mitogenomes of Iberian Romani.

Interesting new insight into the Romani emerged from the analysis of mitogenomes. First, the most distinct lineage of this population is M5a1b1a1. This haplogroup has been observed at a high frequency but low diversity in Romani groups (∼18% according to estimates based on HVS-I segments). The phylogeographic characteristics of M5a1b1a1 (mitogenomes and control region sequences) taken together are compatible with the most likely origin of this lineage being in a proto-Romani population living in Northwest India; further studies will be needed to confirm its possible origin in the Punjab region. This lineage could have left India about 1.5 kya at about the same time as it originated (**Figure 4**), as signaled by the fact that M5a1b1a1 is Indian in origin but it is rare there. Its TMRCA is in good agreement with estimates based on autosomal data for the out-of-India diaspora [20], [22]. From India, M5a1b1a1 carriers could have moved westwards to Pakistan and then to the Middle East, the Caucasus and Europe (**Figure 4**). Clear traces of the origin of M5a1b1a1 and its migration across Europe can be observed in HVS-I sequence databases. The high frequency of this lineage in Europe (and the star-like phylogeny of M5a1b1a1 HVS-I sequences; **Figure 3**), coupled with its low frequency in India, suggests that most likely a population expansion occurred in Europe immediately after the initial diaspora; this hypothesis agrees with inferences based on autosomal data that date the admixture event to about 0.9 kya. [19], [20]. The data also indicate very limited introgression of M5a1b1a1 into East/Central Asia (most likely directly from India). The presence of only a few M5a1b1a1 haplotypes in non-Romani individuals bears witness to the limited genetic exchange between Romani and non-Romani neighboring populations. Thus, M5a1b1a1 was incubated almost exclusively within the Romani and has remained confined to this population since its origin. The phylogeny of M5a1b1a1 is compatible with a rapid and single initial founder event in Europe, as also suggested by studies on autosomal markers [19]. The presence of other South Asian mtDNA lineages in European Romani (other than M5a1b1a1) leaves the door open to the possibility of several out-of-India events; such possibility could be tested by sequencing the mitogenome of other haplogroup M mtDNAs from European Romani individuals. Most of the non-Romani M5a1b1a1 were found in Europe (∼49%), but some appeared in America as well (∼22%), especially in the Rio Grande do Sul state of Brazil. The latter is in agreement with historical documentation that indicates that most of the 19^th^ century Romani overseas migration from Europe travelled from Portugal to Brazil. Curiously, documentation is plentiful concerning the arrival of ‘Ciganos’ (Romani) to the southeastern coast of Brazil [50]; it is documented that the Romani acquired immense fortunes acting as middlemen in the slave trade [51].

Second, Romani carry lineages that could have been incorporated into their mtDNA pool at several geographic locations and times along their out-of-India diaspora westwards to Europe; these lineages were picked up by interactions with neighboring populations. A few of these lineages were found almost exclusively among the Romani; some of them have their origins in the Middle East and Europe but once incorporated into the nomadic Romani, they remained mainly confined within the group, with very little introgression into neighboring populations. U3 is the most common non-Asian lineage in European Romani. Iberian U3 Romani mitogenomes can be allocated to the sub-clade U3b1c, which has a TMRCA of 0.5 kya. The age of U3b1c indicates a lower bound for the founder event that followed admixture in Europe/Near East. The age of U3b1c is likely to be underestimated given that only Iberian mtDNAs have been sequenced; analysis of more U3b1c mitogenomes from Eastern/Central European Romani will allow to investigate whether this lineage can be traced back to the initial contact between Romani and non-Romani in the Balkans perhaps 1 kya [19]. The global phylogeographic distribution of U3 haplogroup tentatively suggests that Romani U3 represent introgression from the Near East rather than Europe.

Third, the geographic origin of a number of Romani lineages is still uncertain given the limited amount of data available; e.g. those belonging to haplogroup H branches are represented by single genomes and do not have control region motifs that are searchable in other public databases. However, the fact that most of the closest phylogenetic branches of haplogroup H Romani mtDNAs have also been found in Europe suggests that these Romani mtDNAs arose most likely after the arrival of their ancestors in Europe.

**Figure 8:**
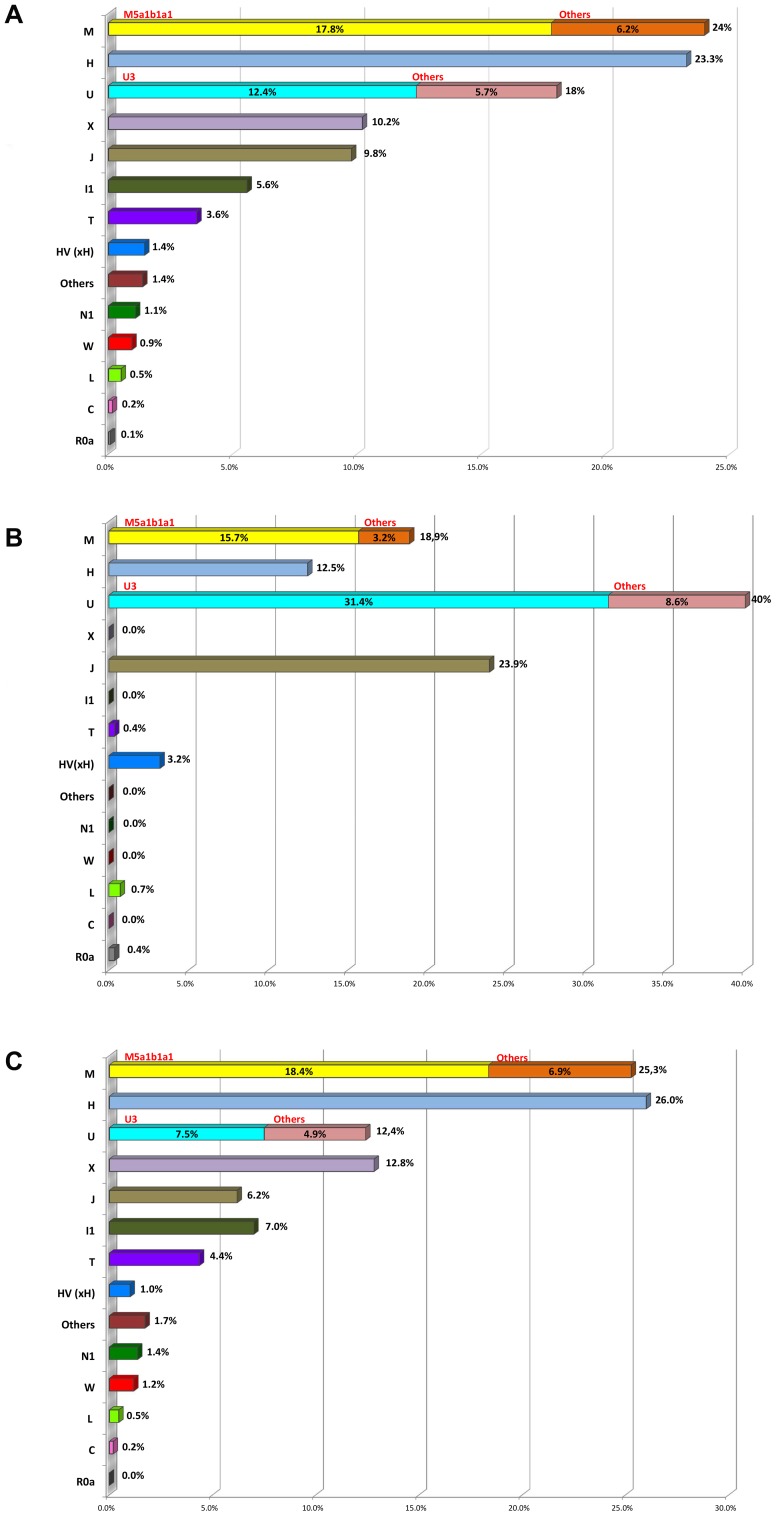
Mitochondrial DNA haplogroup frequencies. (A) European Romani populations; (B) Iberian Romani; (C) European Romani excluding those from Iberia. Note that HV(×H) represents all haplogroups within HV excluding the H branch; L represents all mtDNA clades excluding macro-haplogroups M and N; and the category ‘other’ represents a paragroup that includes all of the haplotypes that could not be unambiguously assigned to any of the other categories considered in the figure.

Fourth, the fact that unrelated Romani from Spain share several haplotypes within different haplogroups (then leading to limited mtDNA variability compared to neighboring European populations) adds further support to the hypothesis that Iberian Romani have kept a low effective population size coupled with relative isolation from non-Romani neighbors after their initial expansion in Europe. This is also compatible with frequent consanguineous marriages among European Romani, a fact that is also observed when examining autosomal SNPs [19]. Further evidence supporting this demographic scenario is the large differences observed in haplogroup frequency patterns between Iberian and Eastern European Romani populations. These are most likely due to genetic drift (**Figure 8**), and they are evident in haplogroups of both Indian (e.g. M5a1b1a1) and European/Near Eastern origin (e.g. X, J, T, U3, X).

Haplogroup M lineages (excluding M1 haplotypes) account for ∼25% of the total mtDNAs in European Romani (most of them belonging to M5a1b1a1; 18%). If we consider that this proportion of mtDNAs originated in India, an upper bound for the European ancestry contribution to the Romani can be estimated at ∼75% on average. This estimate is in good agreement with those based on genome-wide studies (80%; [20]), but is slightly lower, tentatively suggesting that females could have contributed more than males to the preservation of their ancestral Indian legacy. A sub-Saharan contribution is also present in European Romani, but it is only ∼0.5% of their mtDNA genetic pool.

To summarize, the low mtDNA diversity observed in Spanish Romani can only be attributable to a historical demography based on an initial expansion within Europe right after their diaspora from Northeast India ∼1.5 kya, followed by low effective population sizes and isolation (limited maternal gene flow with neighboring populations and endogamic marriages) along their way towards the westernmost edge of Europe. This biological scenario is compatible with their cultural and historical identity.

## Supporting Information

Table S1 **Mitogenomes belonging to haplogroups found in our sample of Iberian Romani or haplogroups phylogenetically closely related.** The list of genomes has been used in **Figures 2**, **5**, **6** and **7**. The new genomes generated in the present study are also included.
(XLSX)

Table S2 **Compilation of control region sequences recorded in the literature in different Romani population samples and those obtained in the present study.**
(XLSX)

Table S3 **List of control region sequences belonging to haplogroup M5a1b1a1 in Romani or non-Romani individuals recorded in the literature, public databases and the present study.**
(XLSX)
